# Continuous outcome logistic regression for analyzing body mass index distributions

**DOI:** 10.12688/f1000research.12934.1

**Published:** 2017-11-01

**Authors:** Tina Lohse, Sabine Rohrmann, David Faeh, Torsten Hothorn

**Affiliations:** 1 Institut für Epidemiologie, Biostatistik und Prävention, Universität Zürich, Zürich, 8001, Switzerland

**Keywords:** Distribution regression, transformation model, conditional distribution, odds ratio, smoking

## Abstract

Body mass indices (BMIs) are applied to monitor weight status and associated health risks in populations.  Binary or multinomial logistic regression models are commonly applied in this context, but are only applicable to BMI values categorized within a small set of defined ad hoc BMI categories.  This approach precludes comparisons with studies and models based on different categories.  In addition, ad hoc categorization of BMI values prevents the estimation and analysis of the underlying continuous BMI distribution and leads to information loss.  As an alternative to multinomial regression following ad hoc categorization, we propose a continuous outcome logistic regression model for the estimation of a continuous BMI distribution.  Parameters of interest, such as odds ratios for specific categories, can be extracted from this model post hoc in a general way.  A continuous BMI logistic regression that describes BMI distributions avoids the necessity of ad hoc and post hoc category choice and simplifies between-study comparisons and pooling of studies for joint analyses.  The method was evaluated empirically using data from the Swiss Health Survey.

## Introduction

Body mass index (BMI) is an anthropometric measure that is relatively easy to capture in epidemiological studies. Thus, it is widely used for describing underweight, overweight, and obesity ^1, 2^. The most prominent standard BMI categories, underweight, normal weight, overweight, and obesity as defined by the World Health Organization [WHO, 3], are commonly applied to ensure comparability and reproducibility of statistical analyses across epidemiological studies ^4, 5^. Such international standards are important for the communication of scientific results, for risk factor assessment and monitoring in populations, and for providing information to the general public. However, categorization of BMI values inevitably leads to information loss because an individual’s weight and height can be measured precisely using simple tools ^1^, but this precision is lost in statistical analyses by such an ad hoc categorization ^6^. The most important problem, however, is the lack of comparability across studies that rely on different categorization schemes. Even more troublesome is the problem of comparability of studies and findings over time because the WHO categories can be expected to be updated to better reflect contemporary BMI distributions. Only roughly half of the studies published up to 2000 that used BMI as a risk factor for death used the WHO categories; the other half relied on a variety of different alternative schemes ^4, 5^. The same problem occurs when the primary interest in a statistical analysis is the comparison of BMI distributions between different risk groups. In this latter situation, we advocate post hoc categorization of model outputs instead of ad hoc categorization of BMI measurements to better combine measurement precision, ease of communication, comparability, and reproducibility. Specifically, we propose that statistical analyses should be based on precise BMI measurements without ad hoc categorization, and then parameters and interesting contrasts thereof should then be categorized post hoc. Such results would be interpretable and universally comparable between studies using any type of category.

### Continuous outcome logistic regression

Conceptually, the traditional approaches to the analysis of BMI can be understood as regression models for the conditional distribution of BMI, given exposure, sex, and covariates ^7– 13^. Treating smoking as the only exposure variable in the following, a generic logistic regression model for BMI, conditional on smoking status, sex, and covariates *x* of the form

                                                logit(ℙ(BMI ≤ *b* | smk, sex, ***x***)) = *r*( *b* | smk, sex, ***x***)           (1)

helps to understand the properties of specific models for BMI and connections between them. Specific classical models, such as binary logistic regression or polytomous logistic regression, are implemented via a specific regression function *r*; details will be given in the next section. The majority of published BMI analyses relied on a small number of ad-hoc cut-off points *b*. After such an ad hoc categorization, only the conditional distribution of BMI at the corresponding cut-off points *b* can be evaluated. The core idea of continuous outcome logistic regression is to model the entire conditional distribution of BMI for all reasonable BMI values simultaneously. This requires that the parameterization of the regression function *r* is a smooth and monotonically increasing function of *b*. The statistical underpinnings of such models were developed only recently ^14, 15^. In models treating BMI as a continuous outcome, the exposure smoking status, sex, and covariates *x* then have an impact on the regression function *r* and thus on conceptually all moments (mean, variance, skewness, kurtosis, etc.) of the conditional continuous BMI distribution. Although such models are more complex, the interpretation of parameters and contrasts thereof remains as simple as in models based on specific categories. For example, the difference between *r*( *b* | former smoker, female, ***x***) and *r*( *b |* never smoker, female, ***x***) is the log-odds ratio of the event BMI ≤ *b* of former female smokers compared to females who never smoked, both of which share the same covariate status ***x***. After traditional ad hoc categorization, this odds ratio can only be evaluated for the small set of cut-off points *b* that define the categories. For continuous outcome logistic regression, the odds ratio can be evaluated for all potential BMI values *b* > 0, which allows the associations for different categorization schemes to be interpreted post hoc. This feature ensures comparability and reproducibility independent of any ad hoc choice of categories.

The continuous outcome logistic regression model can be estimated by maximum likelihood for BMI measurements recorded at different scales ^15^. The likelihood contribution of an individual with a BMI value in the interval ( *b*, *b*] is simply the probability, in light of some specific regression function *r*, of observing a BMI within this interval ^16^

                               ℙ( *b* < BMI ≤ *b* | smk, sex, ***x***)                                        (2)

                                  = ℙ(BMI ≤ *b* | smk, sex, ***x***) – ℙ(BMI ≤ *b* | smk, sex, ***x***)

                                  = expit( *r*( *b* | smk, sex, ***x***)) – expit( *r*( *b* | smk, sex, ***x***)).           

The BMI measurement ( *b*, *b*] can be a narrow numeric interval based on precise measurements of height and weight, or a wide interval corresponding to some standard or non-standard categorization scheme. Thus, continuous outcome logistic regression is applicable to studies that implement different BMI measurement scales or categorization schemes, or even a mixture of those. The procedure thus directly addresses the conceptual problem of lack of comparability between different studies. The aim of our study was to propose a continuous outcome logistic regression model for BMI that is independent of both the BMI measurement scale and cut-offs used for ad hoc categorization, which would allow tailored categorized parameters and contrasts to be extracted, compared, and communicated post hoc. We expected the model to be insensitive to the BMI measurement scales, in light of both the estimated conditional BMI distributions and the covariate model parameters. We evaluated this hypothesis empirically by analyzing the association of smoking status and BMI using data from the Swiss Health Survey 2012 ^17^ while controlling for important covariates, such as age, alcohol intake, diet, physical activity, and socio-economic variables. We compared models fitted to a cascade of increasingly precise BMI values, starting with the four WHO categories and ending with the “exact” BMI values. This allowed an understanding of the impact of the measurement scale on the resulting models. We also expected the results of the novel continuous outcome logistic model for BMI to be comparable to previously reported associations of smoking and BMI, and evaluated this hypothesis for the Swiss Health Survey 2012.

## Methods

### BMI data and models

***Population for empirical evaluation.*** The Swiss Health Survey (SHS) is a population-based cross-sectional survey. Since 1992, it has been conducted every five years by the Swiss Federal Statistical Office ^17^. For this study, we restricted the sample from the 2012 survey to 16,427 individuals aged between 18 and 74 years. Height and weight were self-reported by telephone interview. Records with extreme values of height or weight were excluded (highest and lowest percentile by sex). Smoking status was categorized into never smoked, former smokers, light smokers (1 – 9 cigarettes per day), moderate smokers (10 – 19), and heavy smokers (> 19). Individuals who never smoked stated that they did not currently smoke and never regularly smoked for longer than a six-month period; former smokers had quit smoking but had smoked for more than 6 months during their life. One cigarillo or pipe was counted as two cigarettes, and one cigar was counted as four cigarettes. The following adjustment variables were included: fruit and vegetable consumption, physical activity, and alcohol intake. Information on the number of days per week fruits and vegetables were consumed was available. We chose to categorize as close to the “5-a-day” recommendation as possible ^18^. Fruit and vegetable consumption was combined in one binary variable that comprised the information on whether both fruits and vegetables were consumed daily or not. The variable describing physical activity was defined as the number of days per week a subject started to sweat during leisure time physical activity and was categorized as > 2 days, 1 – 2 days, or none. Alcohol intake was included using the continuous variable grams per day. Education was included as highest degree obtained and was categorized as mandatory (International Standard Classification of Education, ISCED 1-2), secondary II (ISCED 3-4), or tertiary (ISCED 5-8) ^19^. Nationality had the two categories: Swiss and foreign. Language region reflecting cultural differences within Switzerland was categorized as German/Romansh, French, or Italian.

***Models for BMI distributions.*** Binary logistic regression, ordered, and unordered polytomous logistic regression ^20^ were previously applied to the analysis of BMI distributions based on ad hoc categorized BMI values. We will review the corresponding parameterizations and compare the model parameters in the common framework of model (1) before introducing the novel continuous outcome logistic regression for the analysis of BMI distributions.

**Binary logistic regression** For a binary outcome, such as non-obesity vs. obesity (BMI _30_ = *I*(BMI ≤ 30)), the regression function is defined for non-obese individuals only
                                                
*r*(30 | smk, sex, ***x***) = *α*_30_ + **γ**_smk:sex_ + ***x*** ^⊤^ ***β***,
with intercept ***α***_30_, main and interaction parameters **γ** of smoking and sex, and regression coefficients or covariate parameters ***β***. This model evaluates the conditional distribution function for BMI only at *b* = 30. Note that a change of the BMI cut-off point *b* leads to a different model, and thus different parameter estimates for *all* parameters ***α***_*b*_, **γ**, and ***β***. Such models have been reported for *b* = 25 or *b* = 30 ^11, 12^.
**Ordered polytomous logistic regression** This model is also known as proportional odds logistic regression for an ordered categorical outcome, such as the WHO categories ^3^ underweight (BMI _18.5_ = *I*(BMI ≤ 18.5)), normal weight (BMI _(18.5,25]_ = *I*(18.5 *<* BMI ≤ 25)), overweight (BMI _(25,30]_ = *I*(25 *<* BMI ≤ 30)), and obese (BMI > 30). For these four categories, the model is defined by three category-specific regression functions
                                                
*r*(18.5 | smk, sex, ***x***) = *α*_18.5_ + **γ**_smk:sex_ + ***x*** ^⊤^ ***β***
                                                   
*r*(25 | smk, sex, ***x***) = *α*_(18.5,25]_ + **γ**_smk:sex_ + ***x*** ^⊤^ ***β***
                                                   
*r*(30 | smk, sex, ***x***) = *α*_(25,30]_ + **γ**_smk:sex_ + ***x*** ^⊤^ ***β***
or, in more compact notation, by *r*( *b* | smk, sex, ***x***) = ***α***( *b*) + **γ**_smk:sex_ + ***x*** ^⊤^ ***β*** with intercept function

$$\alpha \left(b\right)=\left\{ \begin{array}{l} \;\;\begin{array}{cc} \alpha_{18.5} & \;\;b\leq 18.5 \end{array}\\ \begin{array}{cc} \alpha_{\left(18.5,25\right]} & 18.5<b \end{array}\leq 25\\ \;\begin{array}{cc} \alpha_{\left(25,30\right]} & 25<b\leq 30. \end{array} \end{array}\right.\;\;\left(3\right)$$

The parameters **γ** and ***β*** are the same for all three regression functions and can be interpreted as category-independent log-odds ratios as a consequence of the proportional odds assumption on these parameters. The intercept function increases monotonically. Ordered polytomous logistic regression can be understood as a series of binary logistic regression models where only the intercept is allowed to change with increasing BMI values at cut-off points chosen ad hoc. Self-reported BMI values using the WHO criteria have been analyzed by such a model in 7. The BMI distribution of children categorized at marginal percentiles has been analyzed by a proportional odds model in 13.
An extension of ordered polytomous regression to continuous responses, treating the intercept function *α* as a step-function at the observations with subsequent non-parametric maximum likelihood estimation, was recently suggested by 21. Unlike the model and estimation procedure discussed here, their method does not allow for the different likelihood contributions presented in the next section.
**Unordered polytomous logistic regression** Multinomial logistic regression is equivalent to polytomous logistic regression for an unordered outcome and is a generalization of the proportional odds model as it allows for category-specific parameters **γ**( *b*) and ***β***( *b*) in the regression function
                                                
*r*( *b* | smk, sex, ***x***) = *α*( *b*) + **γ**( *b*) _smk:sex_ + ***x*** ^⊤^ ***β***( *b*)
for *b* ∈ {18.5, 25, 30}. The model can be used to test the proportional odds assumption, *i.e.,* **γ** ≡ **γ**( *b*) and ***β*** ≡ ***β***( *b*) for all *b* ∈ {18.5, 25, 30}. Typically, the model is introduced as a model of the conditional density by the relationship between density and distribution function for discrete variables (as in (2)). This model is very popular for the analysis of BMI-related outcomes ^8– 10^.

The novel continuous outcome logistic regression model can be viewed as a generalization of the above-introduced models from discrete to continuous outcomes. Like these discrete models, the continuous BMI logistic regression model does not require strong parametric assumptions for the conditional BMI distribution, yet it allows to model the conceptually continuous BMI variable by a continuous distribution, regardless of the scale of the actual BMI measurements.

The most important aspect here is a smooth and monotonically increasing intercept function *α*( *b*). In an unconditional model for the marginal BMI distribution

                                     logit(ℙ(BMI ≤ *b*)) = *r*( *b*) = *α*( *b*),

such an intercept function can model arbitrary BMI distribution functions by the term expit( *α*( *b*)) (technical details of the specification and estimation of such an intercept function are given in the Appendix). This essentially removes the need to specify a strict parametric distribution, such as the normal, for BMI. Because of a potential impact of both smoking and sex of the individual on the entire distribution, we stratify this intercept function with respect to these two variables, *i.e.,* one specific intercept function is dedicated to each combination of smoking and sex:

logit(ℙ(BMI ≤ *b* | smk, sex)) = *r*( *b* | smk, sex) = *α*( *b*) _smk:sex_.

This model is also assumption free, because arbitrary BMI distribution functions can be assigned to each combination of sex and smoking.

To facilitate model interpretation, we assume that regression coefficients ***β*** of the remaining covariates are constant across the entire BMI distribution in our final model

                                logit(ℙ(BMI ≤ *b* | smk, sex, ***x***)) = *r*( *b* | smk, sex)     (4)

                                                                                 = *α*( *b*) _smk:sex_ + ***x*** ^⊤^ ***β***.

The regression coefficients ***β*** are log-odds ratios of *all* possible events BMI ≤ *b*, *b* > 0. The interpretation of the parameters ***β*** is the same in logistic regression, proportional odds regression, and the novel continuous BMI logistic regression (4). Of course, these constant regression coefficients might be incorrectly specified. Residual analysis, for example using the residual *U* = ℙ(BMI ≤ *b* | smk, sex, ***x***) for a subject with BMI *b*, can help to detect such misspecifications. Similar to Cox-Snell residuals, the residual *U* is uniform when the model is correct.

Our model (4) can be understood as a joint model of all possible binary logistic regression models for the outcomes BMI ≤ *b* with *b >* 0 under two constraints: (1) the sex- and smoking-level-specific intercept is not allowed to jump abruptly, thus less parameters are required in this joint model, and increases for increasing cut-off points *b*; (2) the regression coefficients ***β*** are held constant as *b* increases. Instead of restricting our attention to specific binary logistic regression models defined by some cut-off points chosen ad hoc, we can answer questions about the odds ratios for all or specific events BMI *≤ b* post hoc based on this model.

The interpretation of the sex- and smoking-specific intercept functions, and thus the associations of smoking and sex with BMI, however, is fundamentally different from the interpretation of the regression coefficients ***β***. Because we allow the entire BMI distribution to change with these two variables in more complex ways, there is no simple interaction term **γ** that captures these parameters in model (4). However, model (4) allows computation of the log-odds ratios for some event BMI ≤ *b* between, for example, female former smokers and females who never smoked for all ***x*** as

*r*( *b* | former smoker, female, ***x***) – *r*( *b* | never smoked, female, ***x***) = *α*( *b*) _former smoker:female_ – *α*( *b*) _never smoked:female_

In this way, the parameters and contrasts we are interested in are not directly parameterized in model (4) but nevertheless can be obtained from this model by relatively simple contrasts. The events BMI ≤ *b* are not restricted to those of a specific categorization of the BMI measurements (such as the WHO categories). Due to the smoothness of the underlying intercept functions, log-odds ratios can be computed for arbitrary BMI values *b* > 0.

***Likelihoods for BMI models.*** Because the regression function *r* is defined for all possible BMI values *b* in model (4), the likelihood (2) can be evaluated for all types of intervals ( *b*, *b*] and also for “exact” BMI values computed as the ratio of weight and squared height. We distinguished between four different likelihood contributions corresponding to four different BMI measurement scales.

**WHO categories (WHO)** The BMI for each individual was reported in one of the four WHO categories corresponding to the intervals ≤ 18.5 (under-weight), (18.5, 25] (normal weight), (25, 30] (over-weight), > 30 (obese). The likelihood contribution of a normal-weight individual is thus
                                                expit( *r*(25 | smk, sex, ***x***)) – expit( *r*(18.5 | smk, sex, ***x***)).
**Other categories (Int 1)** Other studies might have used a different categorization scheme, *e.g.,* the 21 categories defined by BMI intervals for length two:
                                                  ≤ 17, (17, 19], (19, 21], . . . , (35, 37], > 37.                                                
An individual with a BMI value between 19 and 21 thus contributes
                                                expit( *r*(21 | smk, sex, ***x***))–expit( *r*(19 | smk, sex, ***x***))
to the likelihood.
**Numeric intervals (Int 2)** With weight measured in kilogram and height in meters, the BMI is calculated according to its definition as BMI = weight */*height ^2^. However, for an individual 1.75m tall weighting 76kg, all BMI values between 75.5/1.755 ^2^ = 24.51 and 76.5/1.745 ^2^ = 25.12 are consistent with this individual due to rounding error. Thus, this individual contributes
                                                expit( *r*(25.12 *|* smk, sex, ***x***)) − expit( *r*(24.51 *|* smk, sex, ***x***))
to the likelihood, which automatically takes the measurement error into account. These intervals can be expected to be much larger in studies that rely on self-reported weights and heights.
**Exact measurements (Exact)** If extreme precision was used to measure weight and height, BMI = weight */*height ^2^ can be considered an “exact” observation. Because the interval around this value is very narrow, one can approximate the likelihood contribution by the density of the conditional BMI distribution

$$\frac{\partial \text{expit(}r\text{(}b|\text{smk, sex,}\;x\text{))}}{\partial b}\;\left(5\right)$$

evaluated at the “exact” BMI value.

It is important to note that it is possible to evaluate the likelihood when a mixture of these different BMI measurement scales is applied to subsets of the individuals. In subject-level meta analyses, for example, it would be possible to estimate a joint model based on studies using different BMI categorizations or no categorization at all. From a purely theoretical point of view, the application of numeric intervals that take rounding error into account (Int 2) is most appropriate. The remaining three procedures must be considered approximate.

## Empirical results

Comparison of estimated probabilities obtained from the four different likelihoods for model (4) showed that these probabilities were practically identical. For females and males of all smoking categories with baseline covariates, the estimated conditional BMI distribution evaluated at the WHO categories *b* ∈ {18.5, 25, 30} obtained from model (4) are given in Table 1. The model was fitted to BMI observations categorized according to the WHO and to a different categorization with intervals of two BMI units (Int 1). Furthermore, numeric intervals taking rounding error into account (Int 2) and “exact” BMI values were used to estimate model (4). The approximation of the likelihood by the density was very accurate, as the estimated probabilities obtained from models estimated from numeric intervals taking rounding error into account (Int 2) and “exact” BMI values were very close. Differences occurred in the third decimal place if at all. Slightly larger differences were observed between numeric intervals (Int 2) and intervals obtained by categorization Int 1. The more extreme WHO categorization led to the largest differences in these estimated probabilities, but the results were still practically identical.

**Table 1.** Conditional distribution of BMI for WHO Categories. For baseline characteristics *x*, the probabilities obtained from model (4) for BMI ≤ 18.5, BMI ≤ 25, and BMI ≤ 30 are given for each combination of smoking and sex of the individual. The model was fitted using the likelihood (Lik) defined by BMI measurements categorized according to the WHO and according to a different categorization with intervals of two BMI units (Int 1). Numeric intervals taking rounding error into account (Int 2) and “exact” BMI values were used to estimate the model parameters. The differences between these four ways of evaluating the likelihood with respect to the estimated probabilities were marginal.

		BMI:	≤ 18.5				≤ 25				≤ 30			
Sex	Smoking	Lik.:	WHO	Int 1	Int 2	Exact	WHO	Int 1	Int 2	Exact	WHO	Int 1	Int 2	Exact
Female	Never		0.056	0.039	0.043	0.044	0.764	0.735	0.728	0.728	0.943	0.929	0.932	0.932
	Former		0.053	0.038	0.043	0.043	0.748	0.717	0.712	0.712	0.941	0.932	0.931	0.931
	Light		0.079	0.051	0.062	0.063	0.787	0.759	0.755	0.755	0.968	0.955	0.957	0.957
	Medium		0.047	0.042	0.048	0.048	0.768	0.732	0.723	0.723	0.948	0.944	0.942	0.942
	Heavy		0.084	0.086	0.071	0.071	0.740	0.705	0.713	0.712	0.946	0.937	0.938	0.939
Male	Never		0.003	0.004	0.004	0.004	0.546	0.503	0.507	0.507	0.921	0.907	0.910	0.910
	Former		0.000	0.002	0.002	0.002	0.500	0.411	0.405	0.406	0.912	0.887	0.884	0.885
	Light		0.000	0.002	0.003	0.003	0.545	0.497	0.497	0.497	0.932	0.918	0.926	0.925
	Medium		0.000	0.006	0.005	0.005	0.569	0.522	0.521	0.522	0.932	0.914	0.922	0.922
	Heavy		0.006	0.003	0.003	0.003	0.525	0.469	0.462	0.461	0.901	0.881	0.879	0.879

In addition to a comparison of the estimated probabilities, we also compared the proportional log-odds ratios ***β*** among the four BMI likelihoods ( Table 2) and did not find relevant differences. The approximation of the likelihood based on the density resulted in odds ratios numerically almost identical to those obtained from numeric intervals that take the rounding error into account (Int 2). The odds ratios obtained with intervals of Int 1 differed more, but were still negligible. This also applied to the marginally less accurate odds ratios obtained from models fitted to BMI values categorized according to WHO criteria. It should be noted that the lengths of the confidence intervals between the four different BMI likelihoods were in line, which indicated that not only the estimated parameters $\hat{\beta }$ but also their estimated standard errors are comparable among the four approaches. The large sample size led to almost all odds ratios being significant. Age was associated with a shift towards larger BMI values, while higher alcohol intake was associated with marginally reduced BMI. Lower intake of fruits and vegetables as well as less physical activity also indicated a shift to higher BMI values. The BMI distributions of people with a higher education were shifted to the left compared to those of less well-educated people.

**Table 2.** Estimated proportional odds ratios of covariates. The odds ratios exp( $\hat{\beta }$) along with 95% confidence intervals for the covariates age (centered at 40 years), education, alcohol intake, fruit and vegetable consumption, physical activity, education, nationality, and region are given for the four ways of evaluating the likelihood of model (4), *i.e.,,* using BMI measurements categorized according to the WHO and according to a different categorization with intervals of two BMI units (Int 2), numeric intervals taking rounding error into account (Int 2), and “exact” BMI values.

	Likelihood			
Covariate	WHO	Int 1	Int 2	Exact
Age (centered at 40 in y)	0.968 (0.966–0.970)	0.969 (0.967–0.971)	0.968 (0.967–0.970)	0.968 (0.967–0.970)
Alcohol intake (g/d)	1.002 (0.999–1.004)	1.003 (1.001–1.005)	1.003 (1.001–1.004)	1.002 (1.001–1.004)
Fruit and vegetables				
High	1	1	1	1
Low	0.880 (0.824–0.940)	0.928 (0.874–0.986)	0.929 (0.878–0.983)	0.929 (0.878–0.983)
Physical activity				
High	1	1	1	1
Moderate	0.836 (0.774–0.903)	0.850 (0.792–0.912)	0.863 (0.808–0.921)	0.862 (0.808–0.921)
Low	0.695 (0.640–0.756)	0.743 (0.688–0.802)	0.769 (0.716–0.827)	0.769 (0.716–0.826)
Education				
Mandatory	1	1	1	1
Secondary	1.095 (0.992–1.209)	1.252 (1.141–1.373)	1.256 (1.150–1.371)	1.254 (1.149–1.369)
Tertiary	1.604 (1.441–1.786)	1.760 (1.594–1.944)	1.785 (1.625–1.961)	1.781 (1.622–1.956)
Nationality				
Swiss	1	1	1	1
Foreign	0.785 (0.728–0.848)	0.832 (0.776–0.893)	0.810 (0.758–0.864)	0.809 (0.758–0.864)
Region				
German speaking	1	1	1	1
French speaking	1.175 (1.091–1.266)	1.147 (1.071–1.228)	1.134 (1.063–1.208)	1.133 (1.063–1.208)
Italian speaking	1.190 (1.026–1.382)	1.173 (1.024–1.344)	1.236 (1.086–1.405)	1.234 (1.085–1.403)

The BMI values of people of the German-speaking part of Switzerland were higher than those of the French- and Italian-speaking regions.

**Figure 1.**
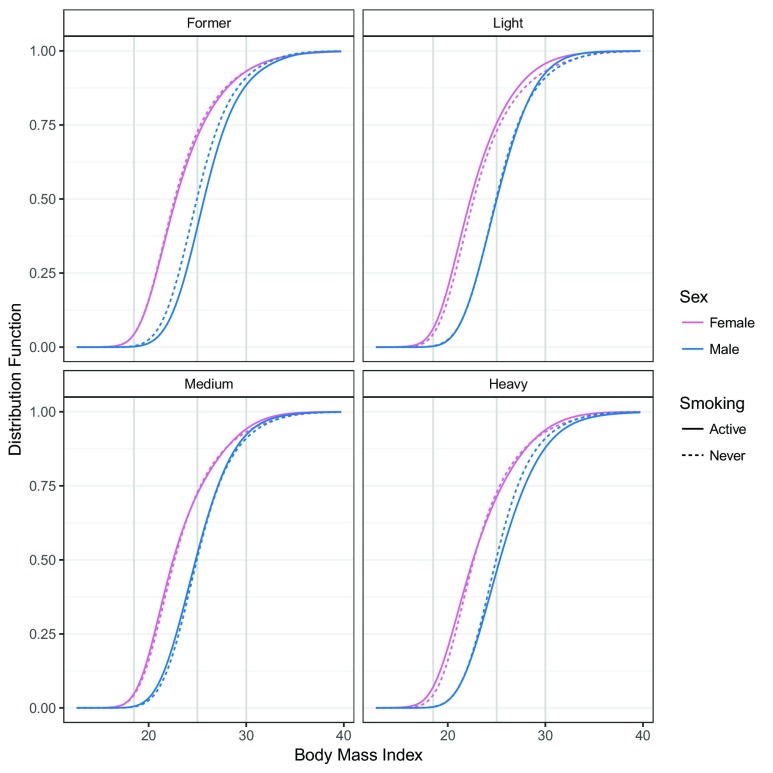
Conditional distribution of BMI. For each combination of smoking and sex, the conditional distribution function of BMI ℙ(BMI ≤ *b* | smk, sex, *x*) corresponding to model (4) was evaluated for baseline covariates *x* at all possible BMI values *b*. Red, female BMI distributions; blue, male BMI distributions; solid lines, BMI distributions of active smokers; dashed lines, never smoked; gray vertical lines, WHO categories 18.5, 25, 30. The model was fitted using “exact” BMI values.

**Figure 2.**
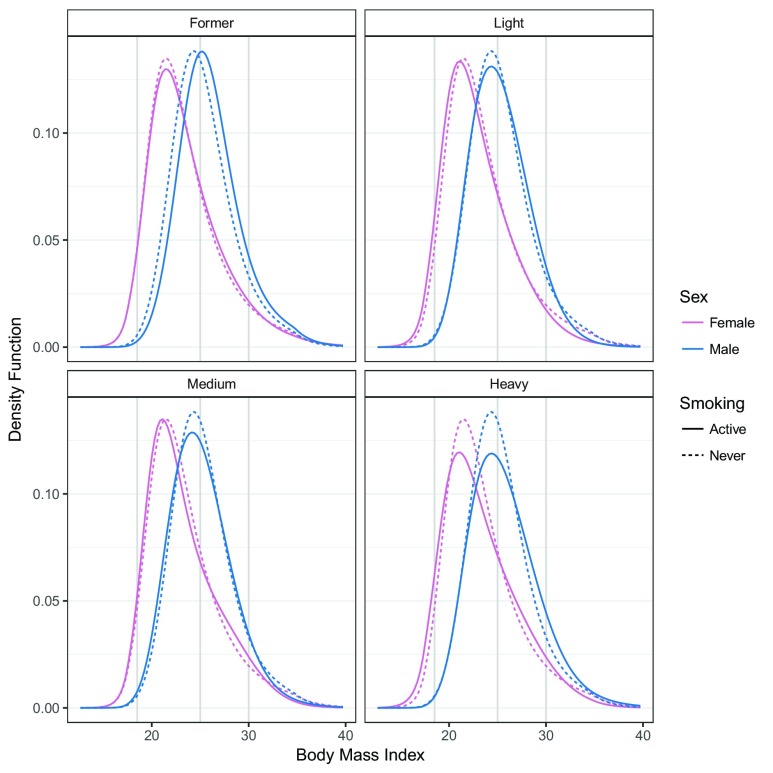
Conditional distribution of BMI. For each combination of smoking and sex, the conditional density of BMI corresponding to model (4) was evaluated for baseline covariates *x* at all possible BMI values *b*. Red, female BMI distributions; blue, male BMI distributions; solid lines, BMI distributions of active smokers; dashed lines, never smoked; gray vertical lines, WHO categories 18.5, 25, 30. The model was fitted using “exact” BMI values.

The estimated conditional BMI distribution for all combinations of smoking and sex were clearly non-symmetric, and the impacts of smoking and sex of the individual related to changes in the mean and higher moments (distribution functions in Figure 1 and density functions in Figure 2). The BMI distribution shifted towards larger BMI values from males who never smoked to male former smokers. In this case, only the mean was affected; the shape of the distribution was constant. The BMI distribution of females who never smoked and female former smokers was similar to those of males. The difference between the two sexes could not be described by a simple shift because the shapes of the two distributions clearly differed. In general, the association of smoking and BMI was less pronounced for females than for males. Compared to the associations of sex ( Figure 1 and Figure 2), the smoking associations were much smaller. We quantified the odds ratios of the smoking association for both sexes for the BMI categories. Table 3 presents the same information as the distribution functions evaluated with the BMI categories (gray vertical lines in Figure 1) on the odds ratio scale in a condensed form. The odds of lower BMI evaluated at BMI ∈ {25, 30} for male former smokers were smaller than for males who never smoked. The odds ratios for underweight and normal weight (BMI ≤ 25) and for non-obesity (BMI ≤ 30) increased for both males and females.

**Table 3.** Estimated non-proportional odds ratios for smoking. Odds ratios comparing all levels of smoking to the level never smoked for the events BMI ≤ 18.5, BMI ≤ 25, and BMI ≤ 30 obtained from model (4) were fitted to “exact” BMI measurements; 95% confidence intervals are given.

		BMI		
Sex	Smoking	≤ 18.5	≤ 25	≤ 30
Female	Never	1	1	1
	Former	0.993 (0.794–1.241)	0.922 (0.825–1.031)	0.987 (0.823–1.183)
	Light	1.462 (1.135–1.884)	1.152 (0.977–1.358)	1.638 (1.187–2.259)
	Medium	1.106 (0.823–1.488)	0.975 (0.830–1.146)	1.182 (0.894–1.564)
	Heavy	1.674 (1.188–2.358)	0.925 (0.756–1.131)	1.116 (0.798–1.562)
Male	Never	1	1	1
	Former	0.457 (0.193–1.081)	0.664 (0.598–0.737)	0.757 (0.649–0.883)
	Light	0.727 (0.275–1.922)	0.960 (0.825–1.117)	1.226 (0.926–1.622)
	Medium	1.352 (0.631–2.900)	1.059 (0.917–1.223)	1.170 (0.911–1.503)
	Heavy	0.852 (0.336–2.161)	0.832 (0.721–0.961)	0.716 (0.579–0.885)

For current smokers, the odds ratio patterns that depended on BMI differed between males and females. All smoking levels were associated with larger odds of being underweight for females and had a U-shaped pattern. For males, this association was reversed and had an inverted U-shaped pattern. In the center of the BMI distribution (BMI ≤ 25), the odds ratios were much closer to 1 for both sexes. The odds ratios for non-obesity (BMI ≤ 30) for females indicated a trend towards smaller BMI values for current smokers. Except for heavy smokers, this effect was also found for males.

## Discussion

Our study showed that it was possible to analyze and compare BMI distributions in terms of standard parameters without the need of ad hoc categorization. Continuous BMI logistic regression, which avoided ad hoc categorization of BMI values, led to deeper insights into the impact of sex of the individuals and smoking status on the continuous BMI distribution. The model results were insensitive to BMI measurement scales or categorization schemes and matched previously reported findings on the impact of smoking and sex of the individuals on BMI. It was obvious from the conditional BMI densities ( Figure 2) that more restrictive models, *e.g.,* a conditional normal distribution with or without sex- and smoking-specific variance ^22^, would describe the BMI distributions less accurately. The corresponding BMI-dependent odds ratios derived from continuous BMI logistic regression ( Table 3) also indicated that a model that assumed proportional and thus BMI-independent odds would not be appropriate because odds ratios varied substantially as BMI cut-off points increased.

We used a parsimonious approach in defining covariate parameters and described the impact of the covariates on the BMI distribution as being linear on the log-odds scale. We therefore assumed that the covariate parameters would be the same in all binary or polytomous logistic regression models regardless of the ad hoc categorization applied. This corresponds to the proportional odds assumption in polytomous logistic regression models. In principle, this assumption could be relaxed by allowing BMI-dependent regression coefficients ***β*** ( *b*), as in multinomial regression. Similar outcome-varying parameters are called time-varying parameters in survival analysis and distribution regression in econometrics ^23, 24^ and are a special case of conditional transformation models ^14^. Ongoing research ^25^ suggests that the assumption of a constant and sex-independent age effect for BMI is oversimplistic and conditional transformation models ^14, 15^, allowing BMI distributions to vary smoothly with age, might provide additional insights.

From a practical point of view, one advantage of continuous outcome logistic regression is the possibility of evaluating the likelihood of BMI values obtained at different measurement scales or using different categorization schemes. This aspect allows the same model to be fitted to data obtained at different scales, and thus allows models from studies using different BMI measurement scales to be compared. The narrower the interval representing the BMI value for a particular individual, the more information is contributed by this individual to the likelihood. In contrast to the common procedure of downscaling all analyses by ad hoc categorization of BMI measurements to the ubiquitous WHO categories ^4, 5^, we propose to fit the same or even joint continuous BMI model to all studies by maximizing a likelihood with measurement-scale specific contributions. In subject-level meta analyses, these likelihood contributions are a mixture of exact, interval, or category-based BMI measurement scales. The likelihood can also be extended to incorporate study-specific left and right truncation when only individuals with BMI values in a pre-defined range are enrolled.

Our findings on the association between smoking and BMI are consistent with the results of previous studies. It has been shown that former smoking is associated with being overweight as well as obesity, especially for males ^8, 9, 11, 12, 26^. Other studies have also observed a positive association of male heavy smokers with obesity, although the association was non-significant when male heavy smokers were compared with males who never smoked ^8, 9^. By contrast, light and moderate smoking was associated with lower BMI values ^8, 9^. In general, current smoking is associated with lower BMI values ^12, 27, 28^. These findings are consistent with previous findings on the effect of smoking on body weight ^29, 30^.

Waiving the need for ad hoc categorization and thus also for agreement on standard categories that define the parameters in models for BMI distributions makes reported scientific results less dependent on these standard categories, and most importantly, less dependent on the WHO criteria. Considering that BMI distributions are subject to change at the population level over time ^2^, insistence on the application of standards defined decades ago leads to an increasing discrepancy between models and data. Continuous BMI logistic regression is an attempt to narrow this gap.

## Appendix: Computational details

The intercept functions *α*( *b*) _smk:sex_ for each combination of smoking and sex were estimated as smooth and monotonically increasing functions of *b*. The constraints expit( *r*( *∞ |* smk, sex, ***x***)) = 1 and expit( *r*(0 *|* smk, sex, ***x***)) = 0 restrict the BMI distribution on the positive numbers. For each of the ten strata given by the five smoking categories and two categories of sex, an intercept function was defined by six increasing parameters of a Bernstein polynomial ^31^ of order five. This choice ensures smoothness and monotonicity and allows flexible intercept functions and thus regression functions *r* and conditional BMI distributions to be described by model (4). The monotonicity constrained on the intercept functions renders the addition of smoothing penalty terms to the likelihood unnecessary, because the effective number of parameters is less than the order of the Bernstein polynomial [see 15, 32, for numerical experiments with varying numbers of parameters]. Simple maximum-likelihood estimation was performed for all model parameters simultaneously. When the likelihood was evaluated for BMI values in WHO categories, the sex- and smoking-specific intercept function was parameterized in terms of the step-function *α*( *b*) (see Formula (3)) defined for the proportional odds model. All computations were performed using R version 3.4.2 ^33^. The **mlt** package ^32, 34^ was used to estimate continuous outcome logistic regression models. The underlying statistical theory is described in 15.

A blueprint for the estimation of conditional BMI logistic regression using the **mlt** package in R, assuming the data are available in a data frame sgb with variables bmi (the numeric BMI values), smoking, and sex (smoking and sex as factors), as well as age and alcohol (numeric age and alcohol intake) with optional sampling weights weights, is


### attach mlt package
library("mlt")
### compute support of BMI distribution
bmis<- quantile(sgb$bmi,
prob=c(.01,.99),na.rm=TRUE)
vBMI<- numeric_var("bmi",
bounds= c(0,Inf),
support= bmis,add=c(-5, 5))
### set-up increasing Bernstein polynomial
bBMI<- Bernstein_basis(vBMI,order=5,
ui="increasing")
### set-up dummy encodings for smoking
### and sex
bSMK<- as.basis(~smoking -1,data= sgb)
bSEX<- as.basis(~sex -1,data= sgb)
### specify the model with strata sex
### and smoking and covariates age
### and alcohol
mod<- ctm(bBMI,
interacting=b(sm= bSMK,sex= bSEX),
shifting= ~age+alcohol,data= sgb,
todistr="Logistic")

                    ### fit model to data with weighted #
### ‘exact’ likelihood
fmod<- mlt(mod,data= sgb,scale=TRUE,
weights= sgb$weights)
### plot conditional BMI distribution for
### 18 year-old never-smoking non-drinking
### female
nsf18<- data.frame(
sex=factor(c("Female","Male"))[1],
smoking=factor(c("Never","Former",
"Light","Medium", "Heavy"))[1],
age=18,alcohol=0)
plot(fmod,newdata= nsf18,
type="distribution")



Continuous outcome logistic regression, as a model for a continuous conditional distribution implemented in **mlt**, has a very strong connection to the Cox proportional hazards model, which describes the conditional continuous distribution of a survival time outcome with fully parameterized log-cumulative hazard function ^15, 32^. A Cox model for the conditional BMI distribution could be written as (see 35)

                         cloglog(ℙ(BMI ≤ *b* | smk, sex, ***x***)) = *r*( *b* | smk, sex, ***x***).

In this case, the logistic link in (1) was replaced by the complementary log-log link. In the absence of covariates ***x***, the results obtained from our continuous BMI logistic regression model and a Cox model stratified by sex and smoking would not be affected by this change, because for each combination of sex and smoking, a corresponding equivalent intercept function *α*( *b*) _smk:sex_ (the sex- and smoking-specific log-cumulative hazard in the stratified Cox model) can be found on both the logit and cloglog scales. However, the interpretation of ***β*** changes from proportional log-odds ratios to proportional log-hazard ratios. In contrast to the partial likelihood of Cox models that treat the intercept functions as nuisance parameters, the likelihood for continuous outcome logistic regression is evaluated for fully parameterized intercept functions and all model parameters are estimated by maximum likelihood [similar to 36]. The corresponding monotonicity constraint allows smooth conditional distribution functions to be estimated without adding smoothing parameters to the likelihood ^15, 32^.

## Data availability

Data from the Swiss Health Survey 2012 can be obtained from the Swiss Federal Statistics Office (Email: sgb12@bfs.admin.ch). Data is available for scientific research projects, and a data protection application form must be submitted. More information can be found here http://www.bfs.admin.ch/bfs/de/home/statistiken/gesundheit/erhebungenSupplementary

## Supplementary material

Supplementary File 1: BMI code.
