# Supplementary material for: Continuous outcome logistic regression for analyzing body mass index distributions
Source: F1000Res. 2017 Nov 1;6:1933. [Version 1] doi: 10.12688/f1000research.12934.1 (PMC5721934; doi:10.12688/f1000research.12934.1)
Supplement: Peer review history

# Peer review: Reviewer response for version 1

**DOI:** 10.5256/f1000research.14025.r27514

**Published:** 2017-12-22

**Authors:** Martyn Plummer

**Affiliations:** 1 Infection and Cancer Epidemiology Group, International Agency for Research on Cancer, Lyon, France

This article illustrates the methodology developed by Hothorn *et al* (2014 ^1^, 2017 ^2^) and implemented in the CRAN package mlt (most likely transformation). The methods are applied to a common epidemiological problem: estimating the effect risk factors for overweight and obesity measured in terms of body mass index (BMI).  BMI is typically coded in categories determined by the World Health Organization (WHO), but ad hoc categories may also be used, and other encodings of BMI are possible (e.g. BMI recorded to a given numerical precision, or calculated exactly from the original height and weight data). The proposed continuous outcome logistic regression model is capable of handling all of these different data formats for BMI. This is illustrated using data from the Swiss Health Survey 2012. The BMI data from this survey are encoded in various ways and the results show consistency of results (table 1 and 2). With the exact encoding of BMI, the conditional distribution of BMI given baseline covariates is estimated from a flexible parametric model using Bernstein polynomials (Figure 1, 2) and odds ratios that depend on the cutpoints (referred to as "non-proportional" odds ratios) are also estimated.

 I have only minor comments on the manuscript

 1) Presentation of the non-proportional odds ratios is obviously more complex, but I wonder why a comparison across different encodings was not made for these odds ratios as it was for the risk factors assumed to have proportional odds ratios in Table 2.

 2) In Table 2, the results for the WHO encoding are somewhat different from the other encodings, which tend to be more consistent with each other. Why?

 3) In Table 2 , I would recommend different units for the continuous variables, as the per-unit odds ratios are attenuated towards 1 by the choice of scale, e.g. odds ratios for a 10-year age difference and a single unit of drink (12g in Switzerland, although 10g is more internationally comparable).

 4) The final paragraph of the discussion seems to imply that the WHO cutpoints will changes in the future as prevalence of overweight and obesity increases. This seems unlikely to me as these cutpoints are normative. For example, despite doubts raised about the utility of the current cutpoints in Asian popoulations, WHO recommends to continue to use them for international comparisons. Having said that, it is possible that the categories will become more detailed in the future as epidemiologists use the three classes of obesity (30-35, 35-59, 39+) and/or break down the "normal" category into two (18.5-23, 23-25).

I confirm that I have read this submission and believe that I have an appropriate level of expertise to confirm that it is of an acceptable scientific standard.

## References

[1] : Conditional transformation models. *Journal of the Royal Statistical Society: Series B (Statistical Methodology)*.2014;76(1) : 10.1111/rssb.12017 3-27 10.1111/rssb.12017

[2] : Most Likely Transformations. *Scandinavian Journal of Statistics*.2017; 10.1111/sjos.12291 10.1111/sjos.12291

# Peer review: Reviewer response for version 1

**DOI:** 10.5256/f1000research.14025.r27859

**Published:** 2017-12-07

**Authors:** Nikolaus Umlauf

**Affiliations:** 1 Department of Statistics, University of Innsbruck, Innsbruck, Austria

The paper "Continuous outcome logistic regression for analyzing body mass index distributions" presents a new approach for estimating conditional BMI probabilities, which avoids the necessity to specify ad hoc BMI categories/cutoff points, e.g., as defined by the World Health Organization. More precisely, the authors use recently developed conditional transformation models that directly model the distribution function of BMI outcomes and are therefore capable to estimate all moments of the response distribution simultaneously. Although computationally the model is quite complex, the authors show in numerous examples that probability statements can be easily obtained in a simple fashion, similar to commonly used models, e.g., when using ordered logistic regression models for BMI categories. The paper is excellently written and clearly shows how information loss can be avoided by the presented model in contrast to applying models onto ad hoc categorized BMI measurements. I only have minor points:

 * In section "Likelihoods for BMI models" the authors show how  likelihood contributions are obtained using different measurement scales. This was immediately clear to me, however, in the next section  the sentence:

   "The model was fitted to BMI observations categorized according to the WHO and to a different categorization with intervals of two BMI units (Int 1)."

   is confusing. What did the authors do exactly, please clarify.

 * In section "Discussion" the authors say that a conditional normal distribution with covariate specific variance would describe the BMI distributions less accurately. I agree, however, using distributions other than the normal might lead to equivalent results. It would be nice to add some more discussion on that.

 * In Table 2 there is a little typo, two commas "i.e.,,".

 Regarding the Data Availability, I think to ensure full reproducibility readers should also be able to download the data from F1000Research, that is why I answered "NO". However, this point is not too critical, since it is possible to obtain the data on request.

I confirm that I have read this submission and believe that I have an appropriate level of expertise to confirm that it is of an acceptable scientific standard.

# Peer review: Reviewer response for version 1

**DOI:** 10.5256/f1000research.14025.r27963

**Published:** 2017-11-29

**Authors:** Noora Kanerva

**Affiliations:** 1 Department of Public Health, University of Helsinki, Helsinki, Finland

The manuscript “Continuous outcome logistic regression for analyzing body mass index distributions” is a very well written paper. The issue is interesting and provides strong suggestion for future studies to consider continuous logistic regression instead of traditional logistic regression. I have only some minor questions/suggestions for the authors:

 1. The main research question was defined clearly in the end of Introduction section (association between smoking and BMI). However, it was less clearly explained why association between smoking and BMI was presented at different levels of sex in such detail in the results, but not the other covariates (e.g., consumption of fruits and vegetables)?

 2. In the discussion (page 8), the authors state “The corresponding BMI-dependent odds ratios from continuous BMI logistic regression (Table 3) indicated that a model that assumed **proportional** and thus BMI-independent odds ….”. Now, looking at the heading of the Table 3, it says “Estimated **Non-proportional** odds ratios for smoking”. Could you please clarify, whether the Table 3 presents results for proportional or non-proportional odds ratios?

 3. To demonstrate the difference between “traditional” and this “new” logistic regression approach, I would like to see also estimated odds ratios for traditional logistic regression with ad hoc categorization for this cohort. Would it be possible to add these results as supplemental material and discuss the differences between these results shortly? I understand that comparing these models was not the aim of this study, but still I think it would be interesting for the readers who are not familiar with this new approach to see this difference.

 4. In the discussion on page 9, the authors compare their results to previous studies. It would be also interesting to compare the magnitude of the association between smoking and BMI in their study and in the previous studies. If there are earlier studies from Switzerland or neighboring countries, are the results more similar with these compared to studies conducted in other, geographically more distant populations (e.g. USA, Asian countries)? Furthermore, it is not clear to me how the similarity of the results obtained using continuous logistic regression and traditional logistic regression should be interpreted. Is it a sign of validity of the new approach or does it imply that the traditional method is as good as the new one?

 5. Please add a paragraph about the possible limitations of the proposed new logistic regression to the Discussion section.

 6. What are the practical implications / future steps based on your findings? Should further studies move from traditional regression model to using this new approach or should the method still be tested in other data?

I confirm that I have read this submission and believe that I have an appropriate level of expertise to confirm that it is of an acceptable scientific standard.
